# Sex-Related Differences in Hip Kinematics During General Movements in Early Infancy: A Biomechanical Cross-Sectional Study

**DOI:** 10.3390/children12050651

**Published:** 2025-05-19

**Authors:** Lucía Fernanda Flores-Santy, Barbara Martina Trujillo Gutiérrez, Cristina Mileny Campaña Iza, Juan Pablo Hervás Pérez

**Affiliations:** 1 MOVS Research Group, Pontificia Universidad Católica del Ecuador, Quito 170525, Ecuador; 2 Carrera de Fisioterapia, Pontificia Universidad Católica del Ecuador, Quito 170525, Ecuador; bmtrujillog@puce.edu.ec (B.M.T.G.); cmcampanai@puce.edu.ec (C.M.C.I.); 3 Faculty of Health Sciences, HM Hospitals, University Camilo José Cela, Urb. Villafranca del Castillo, Villanueva de la Cañada, 49, 28692 Madrid, Spain; jphervas@ucjc.edu

**Keywords:** infant neuromotor development, hip abduction range of motion, General Movements Assessment, kinematic analysis, early motor patterns

## Abstract

The General Movements Assessment provides early insight into motor development’s range of motion; however, its relationship with joint kinematics, such as hip abduction range of motion, remains underexplored. This study analyzed hip abduction kinematics during General Movements, evaluating potential sex differences and variations in movement patterns (Fidgety vs. Writhing), and aimed to provide quantitative data that complement qualitative pediatric assessments. This cross-sectional observational study analyzed video recordings of spontaneous motor activity in 32 infants under three months of corrected age. Hip abduction range of motion was extracted using biomechanical analysis during General Movements. Interrater reliability was evaluated using Fleiss’s Kappa. Correlations were assessed using Pearson’s test, and a two-way ANOVA examined the effects of sex and the type of movements on range of motion. Interrater reliability for movement classification was excellent (Kappa = 0.909, *p* < 0.001). No significant correlations were found between sex or General Movements type and hip abduction range of motion (*p* > 0.68). Two-way ANOVA showed no significant effects of sex, movement pattern, or their interaction on range of motion in either hip (right: *p* = 0.726, left: *p* = 0.823), with small effect sizes (η^2^ < 0.013). A minor asymmetry favoring the right hip was observed but was not clinically significant. Sex and General Movements type did not significantly influence hip abduction range of motion in infants under three months. Early joint mobility appears consistent across sexes and movement patterns, supporting its reliability as a biomechanical marker of typical development.

## 1. Introduction

General Movements (GMs) represent spontaneous and complex body movements observed in fetuses and continue to be observed in infants until approximately 20 weeks of corrected age [1]. These movement patterns serve as early indicators of optimal nervous system functionality, with their quality, variability, and specific characteristics reflecting normative motor behavior during the initial stages of life [1,2].

The assessment of GMs in neonates has emerged as the preferred method for evaluating their optimal neuromotor development [3]. Two types of typical movements are clearly defined in the infant without brain injury: Writhing movements (WMs) and Fidgety movements (FMs). WMs are easily observable during the first 6 to 9 weeks post-term, presenting small to moderate amplitude and slow to moderate velocity, characterized by their elliptical shape [4]. On the other hand, FMs are present up to 15 or 20 weeks post-term and are characterized by being meandering, with small amplitude and variable velocity [5].

Two methodologies have been proposed to evaluate the quality of FMs, whose clinical significance is crucial for the early detection of cerebral palsy. Prechtl postulated the GM Assessment (GMA), which focuses on the kinematic characteristics of this movement, classifying the organization of FMs as continuous, intermittent, sporadic, and absent, considering the latter two as atypical, and includes in this model those FMs of exaggerated appearance [6,7]. Conversely, the Hadders-Algra classification emphasizes the comprehensive assessment of the repertoire of FMs in terms of both variation and complexity. This classification delineates four distinct categories: normal-optimal, normal-suboptimal, moderately atypical, and definitely atypical [8,9,10].

In this regard, various methodologies exist for evaluating GMs in depth, which encompass qualitative techniques relying on visual observation of motor patterns [11], as well as quantitative methods employing advanced automated technologies. Those technologies include accelerometers [12], three-dimensional (3D) motion capture systems [13], motion sensors [2], and 3D cameras, all of which serve to facilitate the recognition and comprehensive analysis of motor skills [14].

While GMs are primarily established and examined using predominantly visual qualitative methodologies, the observed patterns may be significantly influenced by anatomical and biomechanical differences related to sex [15]. In adult populations, where these parameters are clearly defined, significant structural differences are observed in the pelvic ring between males and females [16]. These differences impact the mobility of the pelvic joints and the range of motion in different planes of the lower extremities. Consequently, when compared to females, adult males typically exhibit longer femoral necks, increased widening of the femoral canal, greater femoral displacements, higher neck-shaft angles, and reduced femoral anteversion [17].

In the biomechanical analysis of the pelvis, the anteroposterior diameter of the upper pelvic opening is a significant reference point for differentiating between male and female pelvises. A critical subpoint in this assessment is the anterior superior iliac spine (ASIS) measurement. The distance between the ASIS in females is generally wider than in males, which can influence motor development and affect how individuals perform various physical and functional activities related to their motor capabilities [18].

The anatomical differences between men and women significantly influence their biomechanics and gait patterns. In males, the pubic angle ranges from 48° to 81°, while in females, this angle is comparatively wider, averaging 82° and extending from 64° to 100° [19]. This increased amplitude serves a biomechanical purpose by facilitating childbirth and allowing the vaginal canal to expand due to the pelvis’s enlargement and retroversion. This process, combined with the activation of specific muscles, particularly the elevator ani, plays a crucial role in regulating the descent of the fetal head during delivery [20].

Furthermore, notable differences in gait exist between males and females in adulthood. Females display a more pronounced range of adduction and internal rotation at the hip, attributed to their hip distance-to-femur length ratio [21]. The latter is typically shorter in females, who also possess a greater bilateral acetabular distance than males. Consequently, these anatomical traits are not only evolutionary adaptations but also have a fundamental influence on the locomotion and biomechanics of each individual sex [22].

The anatomical distinctions and biomechanical variances between males and females may considerably influence the amplitude of hip joint movement during the early stages of development. However, despite the potential significance of these differences, existing research has centered mainly on broad descriptions of motor development, frequently overlooking a comprehensive analysis of sex differences and their consequences for motor task performance during this developmental phase [1,2,3,5,7,8].

The examination of hip abduction range of motion (RoM) in infants under three months of age is a crucial aspect in evaluating early neuromotor development, particularly in the context of GM, which acts as a predictive indicator of neurological integrity [23]. As a biomechanical parameter, hip abduction not only reflects joint and muscle health but may also serve as an early indicator of pathological conditions such as developmental dysplasia of the hip, a disorder where early detection is crucial for preventing long-term orthopedic complications [24].

The justification for exploring this range of motion (RoM) within the context of general movement (GM) is based on the understanding that these spontaneous movements represent coordinated patterns arising from the developing central nervous system. Subtle variations in abduction amplitude may be associated with neuromotor dysfunctions or musculoskeletal restrictions of a muscular or articular nature, which might not be detected through conventional examination methods. Moreover, since GM assessments are generally qualitative, quantifying RoM would furnish an objective criterion that would complement the analysis of GM, thereby improving the sensitivity and accuracy of diagnostic evaluations [25].

This study aims to analyze hip joint abduction RoM during GM, evaluate potential sex differences and variations in movement patterns (FM vs. WM), and provide quantitative data that complement qualitative GMA.

## 2. Material and Methods

### 2.1. Study Design/Ethics

This research was conducted as a descriptive, cross-sectional, observational study examining the kinematic parameters of hip joint abduction range of motion (ABD RoM) during active lower limb movements in infants under three months of age while performing General Movements (GMs). The study protocol was approved by the Human Research Ethics Committee of the Pontifical Catholic University of Ecuador (Approval Code: 2019-61-EO). Before participation, written informed consent was obtained from all participating infants’ parents or legal guardians. The study adhered to the ethical guidelines established in the Declaration of Helsinki, ensuring the protection of participants’ rights, confidentiality, and well-being throughout the research process.

### 2.2. Participants

A total of 32 infants younger than three months of corrected age were included in the study, selected using a non-probability purposive sampling strategy to meet the specific objectives of the research. Eligibility criteria required that participants had no known neurological risk factors and had obtained an APGAR score above eight at both one and five minutes after birth. Infants were excluded if they had experienced respiratory difficulties during delivery or presented with pre-existing conditions, such as genetic or motor disorders, or no prior lower limb pathology, as these could interfere with the evaluation of spontaneous motor activity. The sample comprised both full-term and preterm infants, allowing for broader population representation and increasing the external validity of the results.

### 2.3. Procedure and Video Processing

Initially, GMs were categorized by the type of movement they exhibited. To achieve this, two physiotherapists, trained and certified in the GMA and with over 10 years of experience in baby assessment, analyzed the active movements in videos of the subjects and classified them into WM and FM. The two primary evaluators made their decisions independently. A third physiotherapist, trained and qualified in GMA, was available to clarify definitions or classification criteria for these movements. However, this third expert did not directly influence the original evaluators’ ratings, serving solely as a facilitator rather than an additional assessor.

The video was captured by a 23 MP, 24 mm wide HDR camera. The recording protocol for the evaluation was followed as determined by the GMA [7], with no external stimuli that could modify movement, nor wires or skin markers attached to or marked on their extremities. All children were evaluated with bare lower limbs, which allowed for the identification of each joint later.

The resources were then processed using the open-access Kinovea^®^ biomechanical analysis system [26]. This application enables the processing of 2D video images by analyzing kinematic and kinetic variables. In this sense, the kinematic variable of articular amplitude was extracted at the reference points of the hip joint in the lower limbs, using the anatomical points described in the literature for evaluating ranges of motion and goniometry [27]. The reference point of the goniometer axis was placed on the ASIS of the hip side being measured, with its fixed arm projected perpendicular to the imaginary line that connects the contralateral ASIS, and its mobile arm aligned with the femoral diaphysis toward the center of the patella in each lower limb. This setup allowed for the identification of the RoM for abduction during the active movement of the lower limbs in this plane and anteroposterior view exclusively. Once the reference points were positioned, the hip abduction trajectory was tracked in each frame during the GM execution.

### 2.4. Statistical Analysis

The statistical analysis was conducted using IBM SPSS Statistics version 28.0. First, the inter-assessor reliability of the movement classification within the GMA application was analyzed using the Fleiss Kappa index. The Shapiro–Wilk test was applied to assess the normality of the distribution of the continuous variables, with a significance level set at *p* < 0.05. Descriptive statistics were used to summarize the kinematic variable of hip ABD RoM, including the mean and standard deviation.

Pearson’s correlation statistic was used to analyze the relationship among hip ABD RoM, the infant’s sex, and type of GM. This correlation analysis complemented the group comparison by identifying patterns of covariation across joints, providing further insight into the bilateral coordination of spontaneous lower limb movements in early infancy.

To explore the effects of sex and type of GM (WM vs. FM) on hip ABD RoM, a two-way analysis of variance (ANOVA) was conducted. This approach enabled us to assess the main effects of sex and movement pattern, as well as the interaction effect between sex and movement pattern on the outcome variable.

Additionally, the effect size for each main effect and interaction was calculated using eta-squared (η^2^) to determine the magnitude of influence of each factor. Statistical significance was set at *p* < 0.05 for all tests.

## 3. Results

### 3.1. Frequency Statistics

**Figure 1:**
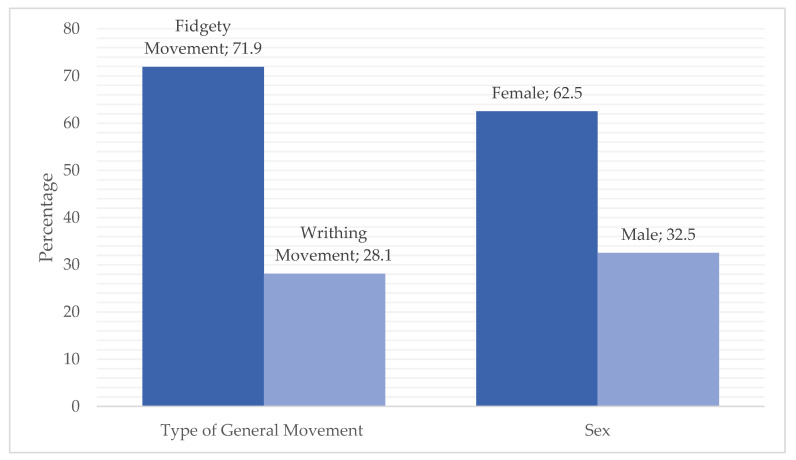
Frequency statistics sex and type of GM.

The frequency statistics showed that the sample consisted of 62.5% male and 37.5% female participants. Concerning the type of GM, 71.9% of the sample exhibited FMs, while 28.1% demonstrated WMs (Figure 1).

### 3.2. Interrater Reliability

The Fleiss Kappa index showed statistically significant interrater reliability for the General Movements Assessment (GMA) video classification (Kappa = 0.909, *p* < 0.001), indicating an almost perfect level of agreement among evaluators. The normality of the data was assessed using the Shapiro–Wilk test, and the homogeneity of variances was verified with Levene’s test. Statistical significance was set at *p* < 0.05, which supports the use of parametric methods in subsequent analyses.

### 3.3. Laterality and Overall RoM Patterns

Central tendency analysis revealed a consistent asymmetry, with a slightly greater right hip RoM (58.36°) compared to the left (55.31°), representing a mean difference of 0.79°. Maximum RoM values were higher in females (right: 80.37°, left: 79.58°) than in males (right: 76.46°, left: 76.63°), which aligns with the higher dispersion observed in girls.

### 3.4. Correlation Analyses

Pearson’s correlation analyses by groups confirmed the absence of statistically significant associations between sex and hip RoM (right: r = 0.069, *p* = 0.707; left: r = −0.074, *p* = 0.688) or between GM classification and hip RoM (right: r = −0.073, *p* = 0.693; left: r = 0.053, *p* = 0.772). All correlations were trivial in magnitude (|r| < 0.08) (Table 1).

Although minor lateral differences in hip mobility were observed favoring the right side, neither sex nor GM classification significantly influenced hip ABD RoM in this cohort of infants. The results suggest that early hip joint mobility demonstrates consistent biomechanical coupling, irrespective of sex or movement quality, supporting the notion that these factors are not reliable predictors of hip RoM in early infancy.

### 3.5. Effects of Sex and General Movement Type on Hip Abduction Range of Motion

A two-way analysis of variance (ANOVA) was conducted to evaluate the effects of sex, GM type, and their interaction on the RoM of hip ABD in both the right and left lower limbs. The results showed no significant main effects of sex or GM type, nor a significant interaction effect between these factors on hip abduction RoM in either limb (all *p* > 0.05) (Table 2).

**Figure 2:**
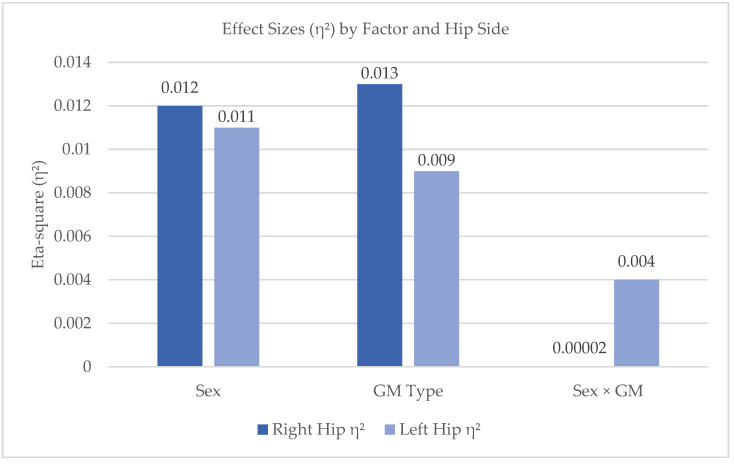
Effect sizes (η^2^) for the factors sex, GM type, and their interaction on hip ABD RoM.

To quantify the magnitude of the observed effects, eta-squared (η^2^) values were calculated. For the right hip, η^2^ values were 0.012 for sex, 0.013 for GM type, and <0.001 for the interaction. For the left hip, η^2^ values were 0.011 for sex, 0.009 for GM type, and 0.004 for the interaction (Figure 2).

These findings suggest that sex and GM pattern do not significantly influence hip abduction RoM in this group of infants under three months of corrected age.

## 4. Discussion

This study aimed to analyze hip joint ABD RoM during GM, evaluate potential sex differences and variations in movement patterns (FM vs. WM), and provide quantitative data that complement qualitative GMA in infants under three months of corrected age using a quantitative biomechanical approach. The findings indicate that neither sex nor GM type significantly impacted hip RoM, and the observed differences between the right and left limbs were minimal, suggesting overall biomechanical symmetry at this early stage of neuromotor development [28].

The almost perfect interrater reliability observed for the General Movements Assessment (Fleiss’s Kappa = 0.909, *p* < 0.001) reinforces the methodological robustness of GM classification in video-based analysis. This level of agreement aligns with previous findings in the literature [29,30], supporting the utility of GMA as a reliable clinical tool even when applied by trained raters using offline recordings.

Although descriptive analyses revealed slightly greater right-side RoM and a tendency for females to display broader movement ranges, these differences were not statistically significant, as confirmed by both Pearson’s correlations and two-way ANOVA. The small effect sizes (η^2^ < 0.013 across all comparisons) suggest that any apparent variations are likely due to typical developmental variability rather than systematic influences related to sex or movement type.

Although not significant, the small effect size observed for GM type in the right hip suggests a potential influence of movement quality on joint kinematics. This coincides with prior findings that associate the Fidgety phase of GM with greater motor complexity and variability, potentially facilitating broader joint excursions [23,31].

On the other hand, the absence of sex-related differences in RoM during early infancy aligns with studies indicating that biomechanical dimorphisms—such as differences in joint laxity or muscle tone—are generally more prominent in later stages of development [32]. At this early stage (<3 months corrected age), musculoskeletal structures and motor patterns are largely shaped by neurological maturation, which is expected to be relatively similar across sexes, particularly in neurologically healthy infants [6]. Moreover, the lack of interaction between sex and GM type suggests that the pattern of movement expression occurs independently of sex at this age, supporting the robustness of the GMA across demographic variables [3,33].

The lack of significant associations between GM type (FM vs. WM) and hip RoM also provides insight on the independence of motor quality and joint RoM at this stage development. While FMs are generally associated with typical neurological outcomes, such as those affecting the lower limbs [34], their presence in this cohort was not linked to increased hip abduction. This suggests that qualitative aspects of movement may not directly correspond to joint-specific kinematic metrics.

According to our findings in the Pearson correlation coefficient, the relationship between joint RoM in the hip and sex is minimal, as the kinematic movements of humans are primarily affected by aging rather than sex differences [35]. For instance, an increase in stride time fluctuation indicates a notable change in the motor production of gait, which is significantly influenced by the aging process [36]. This phenomenon may also be related to changes in the muscle component, as the loss of muscle mass and strength with age, particularly in the muscles responsible for leg flexion and extension, can compromise the efficiency and control of movement during the stride in future stages [37].

This could be due to the activities performed by infants at this stage of their lives, which do not have the motor and environmental requirements that would lead to significant differences in active development movement. However, across the neurodevelopment process, these requirements are significantly influenced by biological sex due to differences in anatomy, physiology, and hormone levels [38]. Therefore, in infants—since they have not been exposed to distinct training stimuli during their first months of life, and their environmental and sensory–motor demands are similar regardless of sex—these kinematic changes are not observed in the studied population.

From a clinical perspective, this finding contributes to the growing body of evidence supporting GMA’s generalizability and its potential for early detection of neurodevelopmental impairments [39]. However, they do not reliably predict sex-based differences in hip joint RoM during spontaneous movement, regardless of sex.

Despite the methodological rigor applied, this study has certain limitations that must be acknowledged when interpreting the findings. First, the small sample size limits the generalizability of the results and decreases the statistical power to detect subtle differences between groups, particularly regarding sex and GM type. Additionally, the cross-sectional design limits inferences about developmental change over time, and the modest sample size may restrict the detection of small effect sizes or subtle interactions. Longitudinal research is needed to examine whether early RoM parameters predict later motor outcomes or relate to neurological risk in clinical populations.

Finally, the kinematic analysis was conducted in a two-dimensional framework which, although valid and accessible, cannot accurately capture out-of-plane displacements, thus limiting the comprehensive characterization of joint mobility. Lastly, the decision to focus exclusively on hip abduction, while relevant to early postural control, excluded other joint parameters that could provide a more holistic understanding of motor development. These limitations highlight the need for future studies employing larger samples, three-dimensional analyses, and multivariate approaches to gain a deeper and more precise understanding of motor development during the first months of life.

## 5. Conclusions

This study contributes to the understanding of spontaneous motor activity by quantitatively analyzing the hip ABD RoM in infants under three months of corrected age during GM. The results did not reveal statistically significant sex-based differences in this kinematic parameter despite greater variability in female infants (right hip SD: 21.92° vs. males’ 10.21°). Although the results did not show statistically significant effects of sex, movement pattern, or their interaction, the observed trends suggest that FMs may be associated with slightly greater joint mobility compared to WM patterns. These preliminary findings reinforce the notion that early motor variability, particularly during FM, may reflect more advanced neuromotor organization, independent of sex.

Importantly, the absence of sex-based differences highlights the developmental homogeneity observed in early infancy and supports the clinical utility of the GMA as a robust tool applicable to both sexes. The incorporation of objective biomechanical analysis in this context contributes valuable quantitative data to a traditionally qualitative field and emphasizes the potential for enhancing early detection strategies by combining movement classification with kinematic measures.

Future studies involving larger cohorts, longitudinal designs, and advanced multivariate modeling are recommended to validate and expand upon these findings and to explore how early kinematic profiles relate to long-term neurodevelopmental outcomes.

## Figures and Tables

**Table 1:** Pearson’s correlation analysis.

Variable Pair		r	*p*-Value
Sex	Right Hip ABD RoM	0.069	0.707
	Left Hip ABD RoM	−0.074	0.688
GM Type	Right Hip ABD	−0.073	0.693
	Left Hip ABD	0.053	0.772

ABD: abduction; and RoM: range of motion.

**Table 2:** Two-way ANOVA results for hip abduction RoM by sex and GM type.

Factor	Right Hip F	Right Hip *p*	Right Hip η^2^	Left Hip F	Left Hip *p*	Left Hip η^2^
Sex	1.23	0.277	0.013	0.26	0.613	0.003
GM Type	2.25	0.145	0.024	0.05	0.828	0.001
Sex × GM Type	0.01	0.924	0.0002	0.15	0.703	0.004

GM: General movement.

## Data Availability

The data supporting the conclusions of this article will be made available by the authors on request. The data are not publicly available due to ethical restrictions on sharing personal data.

## Abbreviations

The following abbreviations are used in this manuscript:

GM	General Movements
FM	Fidgety movements
WM	Writhing movements
ASIS	Anterior superior iliac spine
ABD	Abduction
RoM	Range of motion
